# Community practice of using face masks for the prevention of COVID-19 in Saudi Arabia

**DOI:** 10.1371/journal.pone.0247313

**Published:** 2021-02-19

**Authors:** Yaser A. Al Naam, Salah H. Elsafi, Zeyad S. Alkharraz, Othman A. Alfahad, Khalid M. Al-Jubran, Eidan M. Al Zahrani

**Affiliations:** 1 Department of Clinical Laboratory Sciences, Prince Sultan Military College of Health Sciences, Dhahran, Saudi Arabia; 2 Department of Biomedical Technology, Prince Sultan Military College of Health Sciences, Dhahran, Saudi Arabia; 3 College Deanship, Prince Sultan Military College of Health Sciences, Dhahran, Saudi Arabia; VIT University, INDIA

## Abstract

Community face masking is possibly of great value in reducing COVID-19 transmission, especially when universally adopted with high compliance. The aim of this study is to investigate the knowledge, common misconceptions, barriers, and the compliance of the community with the use of face masks for the prevention of COVID-19. A validated questionnaire was administered to the participants through a web link by using various social media. The collected data were statistically analyzed for significant differences according to demographic variables. The average knowledge of face masks and their role in preventing COVID-19 transmission was 95.64%, with no differences among most of the demographical factors. Older groups and females demonstrated a better attitude towards wearing face masks than other groups did (p<0.001). Another significant difference in the participant’s attitude was noticed between the various educational levels, employment, and nationality (p<0.001). Of the total respondents, 88.2% encouraged wearing face masks. Misconceptions about wearing face masks were very low. The frequency of wearing face masks at public places, workplaces, or social gatherings was 87.2%, 80.5%, and 47.5% respectively. There was a significant variation in the compliance with wearing face masks between the various groups based on age, gender, nationality, and employment status (p<0.001). The inconvenience in wearing face masks was reported by 36.3%. Face irritation and ear pain were reported by 70.2% and 43.5%, respectively. The inconvenience of wearing face masks with eyeglasses was reported by 44.3% of those wearing eyeglasses. In general, the study demonstrated a good attitude among participants towards wearing face masks. Although the respondents in the study were aware of the benefits of wearing face masks, the barriers may have decreased their desire to do so. These barriers include difficulty in breathing, discomfort, face irritation, and ear pain.

## Introduction

The Coronavirus disease, a severe acute respiratory syndrome caused by coronavirus 2 (SARS-CoV-2), commonly known as COVID-19, was first diagnosed in China, in December 2019. Since then, it has resulted, as of 20 November 2020, in an expanding pandemic that has caused more than 58 million cases and more than 1.37 million global deaths across 190 countries and territories [1, 2]. Since the detection of the first COVID-19 case in Saudi Arabia on 2 March 2020 until 19 November 2020, there have been 354,208 confirmed cases with 5,710 deaths [3].

COVID-19 is the third emerging coronavirus that has become a catastrophic public health threat worldwide, following the Severe Acute Respiratory Syndrome Coronavirus (SARS-CoV) in 2002 and the Middle East Respiratory Syndrome Coronavirus (MERS-CoV) in 2012 [4]. Due to the similarity of COVID-19 and previous coronaviruses, initial preventive recommendations for healthcare workers advocated the use of masks for the protection against infection. Social distancing and hand hygiene have been the core measures that were firstly adopted at the beginning to reduce the transmission of COVID-19 in the community [5]. Universal masking was then added following the recent revision of the recommended strategies by the WHO and CDC which has become a ubiquitous practice.

It is believed that community-wide face masking may contribute to the control of COVID-19 by reducing the transmission through infected saliva and respiratory droplets from individuals with subclinical or mild infections [6]. There is also evidence that many people are asymptomatic [7–10]. For example, studies in China and Italy have shown that 78% and 50–75% of people with positive molecular tests were completely asymptomatic [9, 10]. Thus, wearing masks by asymptomatic individuals in public was earlier disputed and regarded as not being effective. However, there are great antithetical evidences that show the use of face masks reduces the risks of COVID-19 transmission to a large degree [11–14]. Mandating face mask use in public is correlated with the daily reduction in COVID-19 transmission, which helps in mitigating the spread of the disease [12]. Despite the consistency in the recommendation for the use of face masks by the healthcare providers and symptomatic individuals, it is not recommended for the general public and the wider community [15]. Nonetheless, public mask wearing is now highly advocated, particularly in areas in which there are high levels of community transmission. However, the use of face masks by healthy individuals in the community to reduce the risk of viral respiratory infections remains contentious.

The current available types of masks include medical masks, N95 masks, and non-medical cloth masks [16]. Medical masks are loosely fitted devices worn by the health care workers and infected individuals to reduce the transmission risk of contagious respiratory droplets between individuals during coughing or sneezing. However, depending on the type of face masks, the protection rate varies from 33 to 100% in the process of expiratory emissions [13]. For example, cloth face masks have moderate efficacy in the prevention of the disseminated respiratory infections resulting from particles of the same size or smaller than those of COVID-19 [17]. Therefore, many countries have enforced the use of face masks.

A high degree of community compliance with face masking will maximize the reduction in the rate of infections. There are several possible reasons that decrease the compliance of individuals with wearing face masks during the outbreaks. The most important of which are the lack of knowledge, misconception, appearance, and barriers preventing compliance. Assessment of the community’s compliance in wearing face masks requires information about their knowledge, attitudes, and perceptions, and then, identification and assessment of the barriers preventing compliance. Physical and social discomfort, confusion or misinformation, low perceived susceptibility to COVID-19, and perceptions of identity and autonomy were reported as the main barriers in using face masks [18].

Compliance is highly affected by the individual’s positive perception, which by itself is influenced by knowledge. Measuring the compliance with the mandate of using face masks by the community is of great importance. However, information on the acceptability of the different types of face masks in preventing COVID-19 is scanty and disputed [14].

A few studies have reported on the knowledge, attitude and practice of health workers regarding the use of face masks for the prevention of SARS-CoV-2 transmission [19, 20]. Studies on the perception and compliance of the community are also lacking. The aim of this study is to investigate the knowledge and compliance of the community in wearing face masks for COVID-19 prevention. The study also investigates the overall perceptions of barriers to wearing face masks. Therefore, possible recommendations for the improvement of community compliance with wearing face masks will be suggested based on the findings of this particular study.

## Materials and methods

This study was approved by the Ethics Review Board of Prince Sultan Military College of Health Sciences, Dhahran (IRB Number IRB-2020-CLS-29), that also approved the lack of parent or guardian consent for children above 16 years. Every participant signed a written informed consent.

For this cross-sectional study, a well-structured, validated, and pretested questionnaire was developed according to the objectives of the study. The questionnaire was developed by a special research group following an extensive review of the literature. The questionnaire consisted of five sections, viz. demographic variables, awareness of the importance of wearing facemasks (5 questions), attitudes and misconceptions towards wearing facemasks (8 questions), barriers to wearing facemasks (5 questions), and finally compliance with wearing facemasks (3 questions). Other questions were related to the types and sources of face masks. A quality assurance question was inserted in the middle of the questionnaire requesting the respondents to choose a specific answer.

A heterogeneous purposive sample of the community who are currently living in Saudi Arabia during the COVID-19 pandemic were included in the study. Questions were validated through a panel of experts before conducting a pilot test of 78 participants, who were not included in the study. Data collected from the pilot test were evaluated for the internal consistency reliability of the questionnaire measured by Cronbach’s alpha, which demonstrated a coefficient value of 0.93. All Saudi residents who were above 16 years old and who had access to the internet were invited to participate. The questionnaire was administered to the participants by a web link through various social media applications and was made available from 21 July to 31 October, 2020.

### Statistical analysis

The participants’ knowledge and attitude were measured by questions on a five-point Likert scale rating, ranging from strongly agree (5), agree (4), neutral (3), disagree (2), and strongly disagree (1). The mean score of every question was calculated out of five. The average knowledge and barriers against using face masking scores were calculated out of 25 points for the five related questions. The attitude and misconception of the participants’ scores were measured out of 40 points for the eight questions. For the questions related to compliance with wearing face masks, the same five-point Likert scale rating was used, ranging from always (5), frequently (4), occasionally (3), rarely (2), and never (1). The average score was calculated out of 15 for the three questions.

Descriptive statistics (frequencies) were completed for all items. The results were analyzed with the use of SPSS software version 20.0 (SPSS, Chicago, Illinois). Internal consistency reliability of the questionnaire was measured by Cronbach’s alpha, where coefficients of ≥ 0.7 demonstrate acceptable internal consistency. We used bivariate correlation between the knowledge, attitude, and compliance with wearing face masks, and one way ANOVA to test the significant differences due to various demographic variables. The statistical significance was set at P < 0.05 for all analyses.

## Results

Out of the 3,824 respondents, 3,572 (93.4%) successfully completed the questionnaire. All of the 252 respondents who failed to correctly answer the quality assurance question were excluded from the study. The various demographical variables of the participants are shown in Table 1. The participants in this study consisted mostly of females (63.4%). A large percentage (79.9%) of the participants were between the ages of 16 and 44, and they were mainly Saudi citizens (94.3%), with a university education (62.5%) and an average household income of 5001–20000 SAR per month (56.5%). Table 2 indicates the mean scores (out of 5) of the knowledge, attitude, and barriers against the use of face masks, while the mean scores of the compliance of the respondents with wearing face masks is shown in Table 3. The average knowledge on when, where, and how to wear a face mask and its role in preventing COVID-19 transmission is 95.64%. Of the total respondents, 88.2% encouraged wearing a face mask while only 5.8% were against doing so. Only 2.8% reported that wearing a face mask might be embarrassing. About 7% agreed that wearing a face mask makes them unattractive and 6.2% reported that wearing a face mask results in an unpleasant appearance. In addition, 46.7% agreed on the difficulty to communicate with a face mask on, and 17.9% reported that their facial expressions might be misinterpreted with their face masks on (Table 2). The mean scores of the frequency of wearing face masks is 4.00 out of 5 (Table 3). The frequency of wearing a face mask in public places, workplaces, or social gatherings was 87.2%, 80.5%, and 47.5% respectively. On the other hand, 4%, 5%, and 26.9% either rarely or never wore face masks in public places, workplaces, or social gatherings, respectively (Table 3).

**Table 1:** Demographic Factors of the total participants (n = 3572).

Variable	Number	Frequency (%)
Age group		
16–24	1579	44.2
25–34	612	17.1
35–44	665	18.6
45–54	463	13.0
55–64	225	6.3
65–74	28	0.8
>75	0	0
Gender		
Male	1309	36.6
Female	2263	63.4
Nationality		
Saudi	3370	94.3
Non Saudi	202	5.7
Region		
Central	1393	39.0
North	247	6.9
East	1040	29.1
South	313	8.8
West	579	16.2
Education level		
Primary School	22	0.6
Elementary School	84	2.4
High School	887	24.8
University	2233	62.5
Postgraduate	346	9.7
Employment		
Employed	1376	38.5
Student	1458	40.8
Retired	295	8.3
Unemployed	443	12.4
Average Monthly Household Income (SAR)		
Below 5000 SAR	486	13.6
5001–10,000 SAR	966	27.0
10,001–20,000 SAR	1056	29.6
20,001–40,000 SAR	561	15.7
40,001–60,000 SAR	187	5.2
More than 60,000 SAR	316	8.8

**Table 2:** Mean scores (out of 5) of the knowledge, attitude, and barriers against the use of face masks (n = 3572).

Questions	SD	D	N	A	SA	Mean ± SD
**Knowledge**						
Face mask reduces the risk of getting COVID-19	0.6	1.1	4.3	30.3	63.7	4.55 ± 0.68
I know when to wear a face mask	0.0	0.6	2.1	29.5	67.7	4.64 ± 0.56
I know how to wear a face mask	0.2	0.3	1.3	26.4	71.8	4.69 ± 0.53
Health experts recommend face mask	0.3	0.7	3.2	33.0	62.8	4.57 ± 0.62
Face mask is important as it protects other people from getting COVID-19	0.4	1.5	5.1	30.6	62.4	4.53 ±0.70
**Attitude / Misconception**						
I have no risk of getting COVID-19	12.4	26.1	34.9	17.6	9.1	2.8 ± 1.13
I don’t have to worry about COVID-19	16.5	27.1	21.8	22.6	12.0	3.14 ± 1.27
I encourage wearing face mask	52.9	35.3	5.9	3.5	2.3	4.33 ± 0.91
It’s difficult to communicate with face mask on	5.2	24.8	23.3	33.4	13.3	2.40 ± 1.10
People might misinterpret my expressions	19.5	43.6	18.6	12.4	5.5	3.25 ± 1.12
I feel embarrassed with face mask	57.7	35.9	3.5	1.9	0.9	1.52 ± 0.74
Face mask makes me unattractive	46.6	35.8	10.6	4.8	2.2	1.80 ± 0.96
Face mask makes unpleasant appearance	38.4	43.1	12.3	4.41	1.8	1.88 ± 0.91
**Barriers to compliance**						
Face mask makes breathing difficult	10.3	22.3	23.7	31.7	12.0	3.13 ± 1.19
It’s uncomfortable to wear mask	13.7	22.6	22.7	30.2	10.7	3.02 ± 1.23
Mask irritates my face	26.8	43.4	16.2	10.1	3.4	2.20 ± 1.05
Mask causes ear pain	14.9	28.6	14.8	26.7	15.0	2.98 ± 1.32
Mask is inconvenient when wearing eyeglass	19.1	25.2	23.0	17.3	15.5	3.85 ± 1.34

SD = strongly disagree D = disagree N = neutral A = agree SA = strongly agree.

**Table 3:** Mean scores (out of 5) of the compliance with the use of face masks (n = 3572).

Practice	N	R	O	F	A	Mean ± SD
How often you wear face mask at public places	1.5	2.5	8.8	24.0	63.2	4.45 ± 0.87
How often you wear face mask at workplace	2.1	2.9	14.4	32.1	48.4	4.22 ± 0.94
How often you wear face mask at during social gathering	13.1	13.8	25.5	20.5	27.0	3.34 ± 1.35

N = never; R = rarely; O = occasionally; F = frequently; A = always.

The average knowledge and attitude scores of the participants towards wearing face masks according to various demographical variables, together with the practice barriers are displayed in Table 4. The results indicated a similar good knowledge score between all age groups, with an average of 22.9 out of 25.00. Similarly, there were no difference in knowledge among the participants with gender, educational level, employment, and average household income. However, a remarkable significant knowledge difference has been observed within the indigenous Saudi population and the expatriates (P = 0.02).

**Table 4:** Knowledge and attitude scores of the participants towards face masks for SARS-CoV-2 protection according to various demographical variable (n = 3572).

Variables	No.	Average knowledge score out of 25 (95% CI)	P	Average attitude score out of 40 (95% CI)	P value
Age group					
16–24	1579	23.00 (22.90–23.11)	0.654	20.91 (20.73–21.09)	<0.001
25–34	612	22.97 (22.77–23.17)		21.44 (21.13–21.74)	
35–44	665	22.95 (22.78–23.13)		21.32 (21.05–21.58)	
45–54	463	23.08 (22.89–23.26)		21.19 (20.85–21.52)	
55–64	225	22.90 (22.60–23.20)		21.50 (21.1–21.88)	
65–74	28	23.57 (22.90–24.24)		23.25 (22.22–24.28)	
Total	3572	22.99 (22.92–23.07)		21.17 (21.05–21.29)	
Gender					
Male	1309	22.97 (22.84–23.10)	0.610	22.03 (21.83–22.23)	<0.001
Female	2263	23.01 (22.92–23.10)		20.67 (20.53–20.81)	
Nationality					
Saudi	3370	22.97 (22.90–23.05)	0.020	21.13 (21.00–21.25)	
Non Saudi	202	23.35 (23.06–23.63)		21.89 (21.37–22.41)	
Education level					
Primary school	22	23.00 (22.00–24.00)	0.235	20.91 (19.28–22.54)	<0.001
Elementary school	84	23.20 (22.75–23.65)		19.98 (19.3–20.65)	
High school	887	22.90 (22.75–23.04)		20.89 (20.66–21.12)	
University	2233	22.99 (22.90–23.09)		21.14 (21.00–21.29)	
Postgraduate	346	23.21 (22.97–23.44)		22.36 (21.96–22.75)	
Total	3572	22.99 (22.92–23.07)		21.17 (21.05–21.29)	
Employment					
Employed	1376	23.05 (22.93–23.18)	0.148	21.74 (21.54–21.93)	<0.001
Student	1458	23.02 (22.9–23.14)		20.86 (20.68–21.05)	
Retiree	295	22.83 (22.58–23.08)		21.20 (20.83–21.56)	
Unemployed	443	22.83 (22.62–23.03)		20.39 (20.09–20.69)	
Total	3572	22.99 (22.92–23.07)		21.17 (21.05–21.29)	
Average monthly household income					
< 5000	486	22.81 (22.61–23.01)		21.05 (20.73–21.37)	0.175
5001–10000	966	22.94 (22.80–23.08)		20.95 (20.74–21.17)	
10001–20000	1056	23.00 (22.86–23.14)		21.29 (21.0–21.52)	
20001–40000	561	23.16 (22.98–23.34)		21.37 (21.0–21.68)	
40001–60000	187	23.27 (22.9–23.62)		21.35 (20.7–21.95)	
> 60000	316	22.97 (22.75–23.20)		21.12 (20.7–21.51)	
Total	3572	22.99 (22.92–23.07)	0.085	21.17 (21.0–21.29)	

Unlike knowledge, there was a significant variation in attitude between the age groups and gender in favor of the older ones and the female groups, respectively. Other significant differences in the participants’ attitudes were detected between the various educational levels, employment, and nationality (p<0.001), but not with the monthly household income (P = 0.17). The attitude towards wearing a face mask was more positive with advanced education level and among employed individuals and retirees rather than students and unemployed. Expatriates demonstrated a better attitude towards wearing face masks than the indigenous Saudis did (Table 2). The compliance of the study’s participants with wearing masks for COVID-19 protection according to the demographical variables is shown in Table 5. There have been significant variations in the compliance in wearing face masks between the various groups based on age, gender, nationality, and employment status in favor of younger age groups (16–34 years old), males, expatriates, advanced education, and employed individuals/students, respectively (p<0.001). There was no difference in compliance with the monthly household income (P = 0.17).

**Table 5:** Average scores of agreement with barriers and the compliance of the participants with the use of face masks for SARS-CoV-2 protection according to various demographical variable (n = 3572).

Variables	No.	Average barriers agreement score out of 25 (95% CI)	P value	Average compliance Score out of 15 (95% CI)	P value
Age group					
16–24	1579	15.64 (15.45–15.43)	<0.001	11.79 (11.67–11.91)	<0.001
25–34	612	15.52 (15.21–15.83)		12.05 (11.58–12.24)	
35–44	665	14.64 (14.34–14.94)		12.21 (12.03–12.39)	
45–54	463	14.46 (14.13–14.79)		12.18 (11.95–12.41)	
55–64	225	14.12 (13.67–14.58)		12.43 (12.15–12.71)	
65–74	28	13.64 (12.00–15.29)		12.82 (12.15–13.49)	
Total	3572	15.17 (15.04–15.30)		12.01 (11.93–12.09)	
Gender					
Male	1309	15.09 (14.88–15.30)	0.350	12.74 (12.62–12.85)	<0.001
Female	2263	15.22 (15.06–15.38)		11.59 (11.48–11.69)	
Nationality					
Saudi	3370	15.15 (15.02–15.29)	0.320	11.93 (11.85–12.01)	<0.001
Non Saudi	202	15.44 (14.91–15.96)		13.34 (13.05–13.63)	
Education level					
Primary school	22	14.5 (12.57–16.43)	<0.001	11.73 (10.85–12.61)	<0.001
Elementary school	84	13.82 (12.98–14.67)		12.46 (11.95–12.98)	
High school	887	14.79 (14.53–15.04)		11.93 (11.76–12.09)	
University	2233	15.30 (15.14–15.46)		11.90 (11.80–12.01)	
Postgraduate	346	15.68 (15.26–16.11)		12.81 (12.58–13.04)	
Total	3572	15.17 (15.04–15.30)		12.01 (11.93–12.09)	
Employment					
Employed	1376	15.17 (14.96–15.38)	<0.001	12.36 (12.24–12.49)	<0.001
Student	1458	15.68 (15.48–15.88)		11.80 (11.67–11.92)	
Retiree	295	13.95 (13.57–14.33)		12.13 (11.86–12.40)	
Unemployed	443	14.30 (13.96–14.65)		11.53 (11.30–11.77)	
Total	3572	15.17 (15.04–15.30)		12.01 (11.93–12.09)	
Average monthly household income					
< 5000	486	15.02 (14.64–15.41)	0.383	12.04 (11.80–12.27)	0.766
5001–10000	966	15.09 (14.85–15.33)		11.96 (11.80–12.12)	
10001–20000	1056	15.19 (14.96–15.42)		12.01 (11.86–12.16)	
20001–40000	561	15.28 (14.98–15.57)		12.12 (11.95–12.30)	
40001–60000	187	15.71 (15.10–16.31)		11.85 (11.51–12.20)	
> 60000	316	15.09 (14.66–15.51)		12.01 (11.75–12.27)	
Total	3572	15.17 (15.04–15.30)		12.01 (11.93–12.09)	

When the participants were asked about the barriers against wearing face masks, 32.6% agreed that wearing face masks makes it difficult to breathe, while 43.7% disagreed. Discomfort in wearing face masks was reported by 36.3%, while 40.9% disagreed with this. Face irritation and ear pain were reported by 70.2% and 43.5%, respectively. The inconvenience of wearing face masks with eyeglasses was agreed upon by 44.3% of those wearing spectacles.

The majority of the study’s participants (74.8%) used medical masks while cloth masks and N95 masks were used by 9.3% and 2%, respectively. Others (13.7%) reported using mixed types of the masks and 0.3% reported using other types that mainly included the veil or face cover used frequently by females in Saudi Arabia (Table 6).

**Table 6:** Type of mask used by the participants and the percentage between parentheses.

Type	Surgical	Cloth	N95	Mixed	Other
No. (%)	2672 (74.8)	331(9.3)	72 (2.0)	488 (13.7)	9 (0.3)

When asked about the sources of the face masks, most of the respondents (67.1%) said that they got them from pharmacies, while 7.2%, 0.9%, and 0.3% mentioned that they obtained them from supermarkets, workplaces, and online shopping, respectively (Table 7). A large proportion mentioned mixed sources or other sources that included homemade, medical supply store companies, and tailor shops.

**Table 7:** Source of face masks used by the participants and the percentage between parentheses.

Source	Pharmacy	Supermarket	Workplace	Online	Mixed	Other
No. (%)	2398 (67.1)	258 (7.2)	32 (0.9)	11 (0.3)	779 (21.8)	94 (2.6)

A Pearson correlation revealed that there was a positive correlation between knowledge and attitude (r = 0.10, p<0.001), knowledge and practice (r = 0.33, p<0.001), towards the use of face masks for the prevention of COVID-19.

## Discussion

In May 2020, the Ministry of Interior of Saudi Arabia has mandated the wearing of face masks in public places and made them available at a low cost. The regulation included prescribed violation penalties. The current study has revealed a good compliance rate with wearing face masks in public places (87.2%) and workplaces (80.5%). Before the COVID-19 pandemic, the compliance of wearing face masks for the prevention of H1N1 pandemic in 2009 was found to be 8%, 11%, 19%, 63%, and 71% in the USA, UK, Argentina, Japan, and Mexico [21]. A study in Hong Kong reported that face mask compliance was very high, between 95.7% and 97.2% across the regions studied, and that COVID-19 infections in outdoor activity gatherings where masks were not worn, were significantly more common than that in workplace settings [6]. A study in Malaysia detected that only 51.2% complied to wearing face masks during this COVID-19 pandemic [22].

The use of face masks by the community is potentially of high value in reducing community infections of COVID-19 [15, 23 (Van der)]. The community-wide benefits are probably more when face masks are used together with social distancing, and when they are universally adopted with high compliance [15]. Model simulations, using data relevant to COVID-19 dynamics in some US states, suggest that broad acceptance of face masks may markedly reduce community transmission of COVID-19 and decrease morbidity and mortality [15]. In order to reduce the potential risk of the asymptomatic or poorly symptomatic people in COVID-19, community-wide use along of other measures seem to be extremely beneficial. Many studies suggested that the use of a mask by healthy people in the community could be protective for COVID-19, especially when asymptomatic individuals are playing a role in the transmission [3.5, 24].

Despite the controversy and the worldwide mask production sacristy, more countries are moving forward with recommendations or directives to wear face masks in public [2].

The wearing of face masks in this study was found to be significantly associated with factors related to age, gender, region, education, and employment. In fact, younger age groups (16–34 years old), males, expatriates, advanced education, and employed and students showed a greater compliance than other groups. Similar findings have also been reported in another study [25].

Community wearing of face masks seemed to be effective even without other protective measures [11]. Another study indicated that wearing face masks combined with social distancing can be very effective and can significantly reduce the COVID-19 risk in the community [25].

Medical masks were mostly used by the respondents, followed by cloth masks and N95 masks. A few of the respondents reported their reliance on the face cover or veil used frequently by females in Saudi Arabia. Its effectiveness has long been proven in practice, despite it being loose fitting which may cause aerosol droplet leakage [26, 27]. On the other hand, homemade masks, made of cotton fabric, although less effective, have significantly reduced droplet and particle transmission [28]. The most important factor in the selection of the type of mask is its ability to contain the generated droplets [29]. The N-95 type of face mask is more effective than medical masks because of its ability to collect particles only when the mask fits to the face sealing the boundary [29]. Depending on the type of cloth used, the number of layers, and washing cycles, cloth masks provide a protection between 40% and 97% [26]. It has been reported that, in spite of imperfect fit and improper compliance, the use of any type of face mask is likely to decrease the disease exposure and thus the risk of infection at the population level [30].

Wearing masks could be effectively practiced at a low cost without considerably disturbing the social habits [22]. Guidelines for wearing masks differ significantly between countries [22]. It has been reported that the maximum community benefit of using a face mask depends on the compliance and the early use during the transmission together with hand hygiene [11].

A study on a Malaysian community has reported an overall rate of the knowledge of 80.5%, and most participants (83.1%) demonstrated positive attitudes toward the prevention of COVID-19 [25].

The study has demonstrated a good attitude towards wearing face masks. In this particular study, 38.5% of the participants thought that they were at high risk, and 88.2% encouraged wearing face masks. A study done with healthcare workers demonstrated a positive attitude but there was a moderate-to-poor level of knowledge and practice regarding the use of face masks. Therefore, it has been recommended that healthcare worker and general public awareness campaigns be conducted concerning the appropriate use of face masks by using the available social media resources which would be especially helpful during the pandemic [18].

Although this particular study’s respondents were aware of the benefits of wearing face masks, the barriers against doing so may have decreased their willingness to comply.

The compliance with wearing face masks seemed to have been affected by how comfortable mask wearing was, especially for long-term use [31]. However, N95 masks seemed to have been more uncomfortable, demanding tedious cautions [31]. Despite the good compliance and knowledge about the importance and when and how to wear face masks, there were some perceived social barriers against the practice. The stigma associated with wearing a mask has been reported several times [27].

Around 93% of the participants agreed on the anticipated benefits of wearing masks, and most of the participants knew when (97.2%) and how (98.2%) to wear masks. About 32.6% reported that a face mask makes it difficult to breathe, while 43.7% disagreed with this. Discomfort with wearing face masks was reported by 40.9% of the participants. Face irritation and ear pain were reported by 70.2% and 43.5%, respectively. The inconvenience of wearing face masks with eyeglasses was agreed upon by 44.3% of those wearing eyeglasses. Some participants were not satisfied with the experience of mask-wearing. Discomfort including poor fit, ear pain, and difficulty breathing has been reported before [32]. Evidence suggests that the potential benefits of wearing masks are likely to outweigh the potential harms of the spread of Covid-19 within a community [33, 34].

The study indicated positive correlations between the knowledge and attitude as well as knowledge and practice. Therefore, better compliance with wearing face masks requires information about their importance, a positive attitude, and lack of misconceptions. Appropriate knowledge can lead to a positive attitude, resulting in less misconceptions and good practices. Despite the large sample size included in this study, its major limitations were the influence of socially desirable traits on the responses, in addition to the potentiality of recall bias of the participants.

## Conclusions

The study has revealed a good compliance rate in wearing face masks in public and work places. Medical masks were mostly used by the respondents followed by cloth masks and N95 masks. A small proportion of the respondents reported their reliance on the face cover or veil used frequently by females in Saudi Arabia. The study has demonstrated a good attitude towards wearing face masks. Although the study’s respondents were aware of the benefits of wearing face masks, the barriers against doing so may have decreased their willingness to comply. About 32.6% reported that wearing face masks makes it difficult to breathe, while 43.7% disagreed with this. Inconveniency with wearing face masks was reported 40.9% of the participants. Face irritation and ear pain were reported by 70.2% and 43.5%, respectively. Inconveniency when wearing eyeglass was agreed upon by 44.3% of the eyeglass users. Some participants were not satisfied with the experience of mask wearing. The discomfort including ear pain, and difficulty breathing has been reported before.

## Supporting information

S1 Questionnaire (PDF)
