# Supplementary material for: Community practice of using face masks for the prevention of COVID-19 in Saudi Arabia
Source: PLoS One. 2021 Feb 19;16(2):e0247313. doi: 10.1371/journal.pone.0247313 (PMC7894919; doi:10.1371/journal.pone.0247313)
Supplement: S1 Questionnaire — (PDF) [file pone.0247313.s001.pdf]

Community compliance with wearing face masks for the prevention of COVID-19 in Saudi Arabia

*Required

Face masks study

The Covid-19 pandemic has globally impacted how people interact, communicate and work. Wearing face masks has been recommended by health experts globally to combat the spread of the virus. This study examines the experiences and difficulties of face mask wearing faced by the public in various spaces. By choosing I agree, you are agreeing that you are at least 16 years old, that you have read the information about the experiment, and that you voluntarily agree to take part in this study. This study was approved by PSMCHS Institutional Review Board (IRB-2020-CLS-029)

For any concerns, kindly reach: zalkharraz@psmchs.edu.sa

1. *

Mark only one oval.

- ⬭ I agree

Personal Information

2. Age *

Mark only one oval.

- ⬭ 16 - 24
- ⬭ 25 - 34
- ⬭ 35 - 44
- ⬭ 45 - 54
- ⬭ 55 - 64
- ⬭ 65 - 74
- ⬭ 75 or above

3. Sex *

Mark only one oval.

- Male
- Female

4. Nationality *

Mark only one oval.

- Saudi
- Non Saudi

5. Current Education *

Mark only one oval.

- Primary School
- Elementary School
- High School
- University
- Postgraduate
- Other: ______________________

6. Employment *

Mark only one oval.

- Employed
- Student
- Retired
- Unemployed
- Other: ______________________

7. Household monthly income (income of all members of the family) *

Mark only one oval.

- Below 5000 SAR
- 5001 - 10000 SAR
- 10001 - 20000 SAR
- 20001 - 40000 SAR
- 40001 - 60000 SAR
- Greater than 60001 SAR

8. Region *

Mark only one oval.

- Bahah
- Ha'il
- Najran
- Jawf
- Jizan
- Madinah
- Makkah
- Eastern Province
- Northern Borders
- Qassim
- Riyadh
- Asir
- Tabuk

To what extent do you agree with the following statements?

9. Wearing face mask reduces my risk of getting COVID-19 *

Mark only one oval.

- Strongly agree
- Agree
- Neutral
- Disagree
- Strongly disagree

10. I know when I should wear a face mask *

Mark only one oval.

- Strongly agree
- Agree
- Neutral
- Disagree
- Strongly disagree

11. I know how to wear a face mask *

Mark only one oval.

- Strongly agree
- Agree
- Neutral
- Disagree
- Strongly disagree

12. I have a high risk of getting COVID-19 *

Mark only one oval.

- Strongly agree
- Agree
- Neutral
- Disagree
- Strongly disagree

13. I don’t have to worry about COVID-19 *

Mark only one oval.

- Strongly agree
- Agree
- Neutral
- Disagree
- Strongly disagree

14. It’s difficult for others to see my face expressions when wearing a face mask *

Mark only one oval.

- Strongly agree
- Agree
- Neutral
- Disagree
- Strongly disagree

15. People will misinterpret my feelings when wearing a face mask *

Mark only one oval.

- Strongly agree
- Agree
- Neutral
- Disagree
- Strongly disagree

16. I feel embarrassed when wearing a face mask *

Mark only one oval.

- Strongly agree
- Agree
- Neutral
- Disagree
- Strongly disagree

17. Wearing face mask makes me unattractive *

Mark only one oval.

- Strongly agree
- Agree
- Neutral
- Disagree
- Strongly disagree

18. The appearance of face mask is unpleasant *

Mark only one oval.

- Strongly agree
- Agree
- Neutral
- Disagree
- Strongly disagree

19. I have a breathing difficulty when wearing a face mask *

Mark only one oval.

- Strongly agree
- Agree
- Neutral
- Disagree
- Strongly disagree

20. It's uncomfortable to wear face mask *

Mark only one oval.

- Strongly agree
- Agree
- Neutral
- Disagree
- Strongly disagree

21. Face mask irritates my face *

Mark only one oval.

- Strongly agree
- Agree
- Neutral
- Disagree
- Strongly disagree

22. Face mask causes ear pain *

Mark only one oval.

- Strongly agree
- Agree
- Neutral
- Disagree
- Strongly disagree

23. Face mask is inconvenient while wearing eyeglasses *

Mark only one oval.

- Strongly agree
- Agree
- Neutral
- Disagree
- I don't wear eyeglasses

24. Please choose "Neutral" for this question *

Mark only one oval.

- Strongly agree
- Agree
- Neutral
- Disagree
- Strongly disagree

25. Health experts recommend wearing face mask *

Mark only one oval.

- Strongly agree
- Agree
- Neutral
- Disagree
- Strongly disagree

26. Face mask is important because it protects other people from getting COVID-19 *

Mark only one oval.

- Strongly agree
- Agree
- Neutral
- Disagree
- Strongly disagree

27. I don’t need to wear a face mask; other people should take care of themselves *

Mark only one oval.

- Strongly agree
- Agree
- Neutral
- Disagree
- Strongly disagree

How frequent

28. How often do you wear face mask at workplace? *

Mark only one oval.

- Always
- Frequently
- Occasionally
- Rarely
- Never

29. How often do you wear face mask at public places? *

Mark only one oval.

- Always
- Frequently
- Occasionally
- Rarely
- Never

30. How often do you wear face mask at social gatherings? *

Mark only one oval.

- ( ) Always
- ( ) Frequently
- ( ) Occasionally
- ( ) Rarely
- ( ) Never

31. What type of mask do you use? *

Tick all that apply.

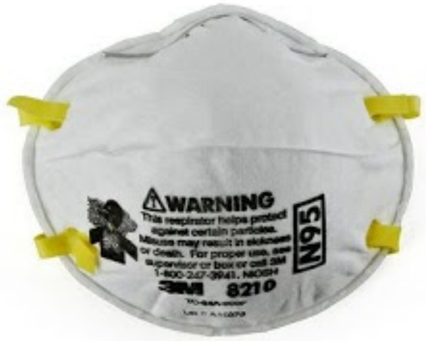

- [ ] N95

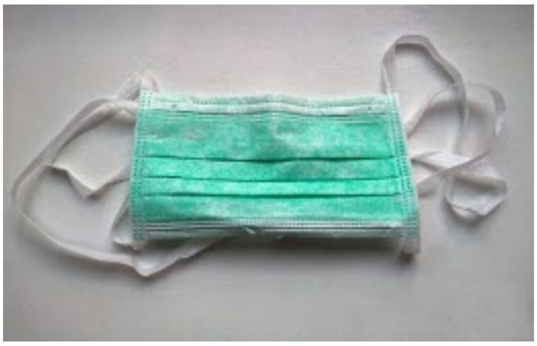

- [ ] Surgical

Other: [ ] ____________

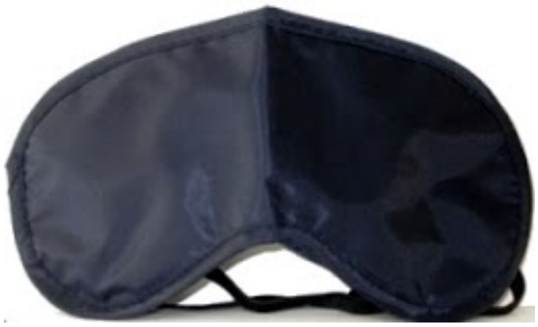

- [ ] Cloth

32. From where do you get your face mask? *

Tick all that apply.

- [ ] Pharmcy
- [ ] Supermarket
- [ ] Tailor shop

Other: [ ] ______________________
